# Targeted next generation sequencing and family survey enable correct genetic diagnosis in CRX associated macular dystrophy – a case report

**DOI:** 10.1186/s12886-021-01919-1

**Published:** 2021-04-09

**Authors:** Saoud Al-Khuzaei, Karl A. Z. Hudspith, Suzanne Broadgate, Morag E. Shanks, Penny Clouston, Andrea H. Németh, Stephanie Halford, Susan M. Downes

**Affiliations:** 1 Nuffield Laboratory of Ophthalmology, Nuffield Department of Clinical Neurosciences, University of Oxford, Oxford, UK; 2 Oxford Eye Hospital, John Radcliffe Hospital, Headley Way, Oxford, OX9 3DU UK; 3 Oxford Medical Genetics Laboratory, Oxford University Hospitals NHS Foundation Trust, Oxford, UK; 4 Oxford Centre for Genomic Medicine, Oxford University Hospitals NHS Foundation Trust, Oxford, UK

**Keywords:** Retina, Macular dystrophy, Next generation sequencing, CRX, ABCA4 sequence variant, mutation, Phenotype/genotype, reduced penetrance, family survey

## Abstract

**Background:**

We present 3 members of a family with macular dystrophy, originally diagnosed as Stargardt disease, with a significantly variable age at onset, caused by a heterozygous mutation in *CRX*.

**Case presentation:**

A 43-year-old female with bull’s eye maculopathy, whose sister was diagnosed with Stargardt disease previously at another centre, was found to have a single *ABCA4* variant. Further examination of the family revealed that the asymptomatic father was also affected, indicating a dominant pattern of inheritance. In addition, the *ABCA4* variant was not identified in the sister originally diagnosed with Stargardt disease. Next generation sequencing identified a heterozygous c.121C > T, p.R41W missense mutation in *CRX* in all 3 affected members.

**Conclusions:**

We describe a common phenotype, but with variable age at onset, with autosomal dominant inheritance and reduced penetrance in a family found to have a pathogenic sequence variant in *CRX*. This illustrates the importance of panel based molecular genetic testing accompanied by family studies to establish a definitive diagnosis.

## Background

Macular dystrophies (MD) may have certain recognisable clinical features that can pinpoint a specific diagnosis enabling a secure phenotype classification. However, in some cases patients present with retinal features that are shared by different conditions, known as phenocopies. In addition, since the advent of NGS, more than one potential causative gene variant may be identified in a family with an inherited retinal dystrophy thus making a definitive diagnosis difficult. However molecular genetic testing, accompanied by careful family studies with segregation of the variant with disease, can enable a conclusive diagnosis. This is important for prognosis, genetic counselling, and potential therapeutic interventions such as gene therapy.

## Case presentation

**Fig. 1:**
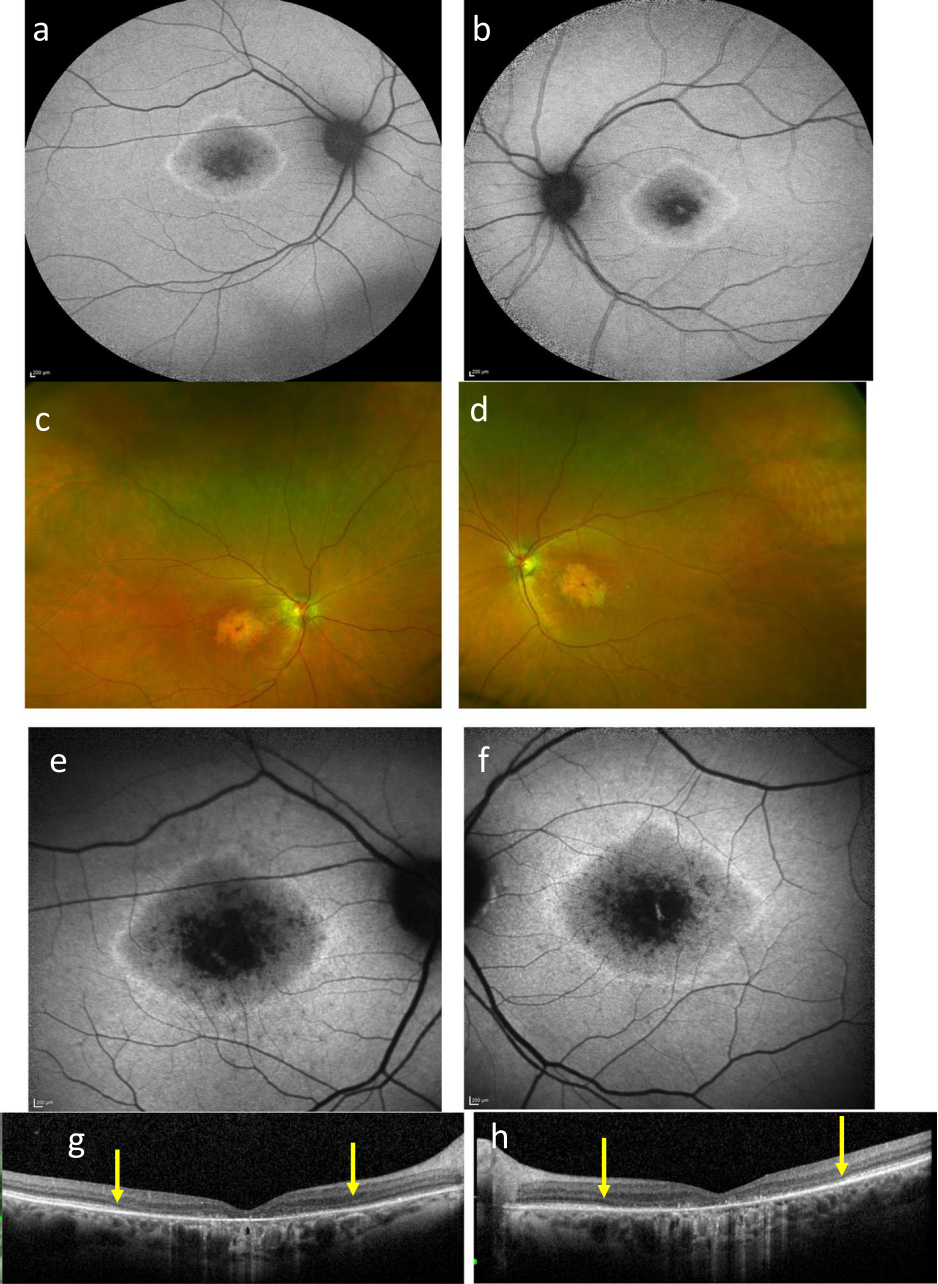
Images of proband at different time points: **a** and **b** show AF at age of 51, 8 years after first presentation with a central preserved spot of AF, surrounded by atrophy, and a ring of AF external to the atrophy. **c** and **d** show wide field Optos images at age of 59 (16 years after presentation) with central atrophy; **e** and **f** show AF at the same time showing enlargement of the decreased AF signal; **g** and **h** OCT showing loss of the EZ between the yellow arrows Key: Autofluorescence AF, Optical coherence tomography (OCT), Ellipsoid Zone (EZ).

**Fig. 2:**
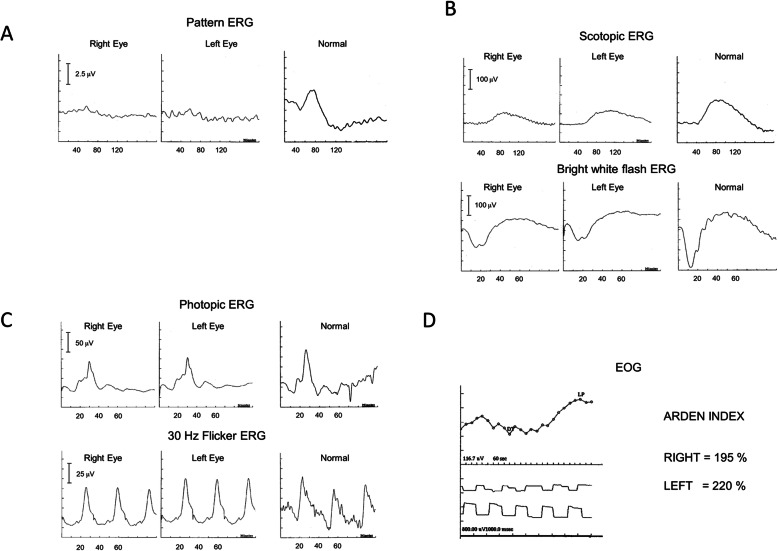
Electrophysiology testing results for the proband (**a**) Pattern ERG with the reduction in amplitude in both eyes (**b**) Scotopic ERG showing increased ‘**a**’ and ‘**b**’ wave implicit times, and reduction in ‘**b**” wave amplitude in both eyes (**c**) Photopic ERG showing normal ‘**a**’ and ‘**b**’ wave latencies and normal ‘**a**’ and ‘**b**’ wave amplitudes for both flash and 30 Hz flicker stimuli (**d**) electro-oculogram showing borderline Arden Index in the right eye and normal in the left eye

**Fig. 3:**
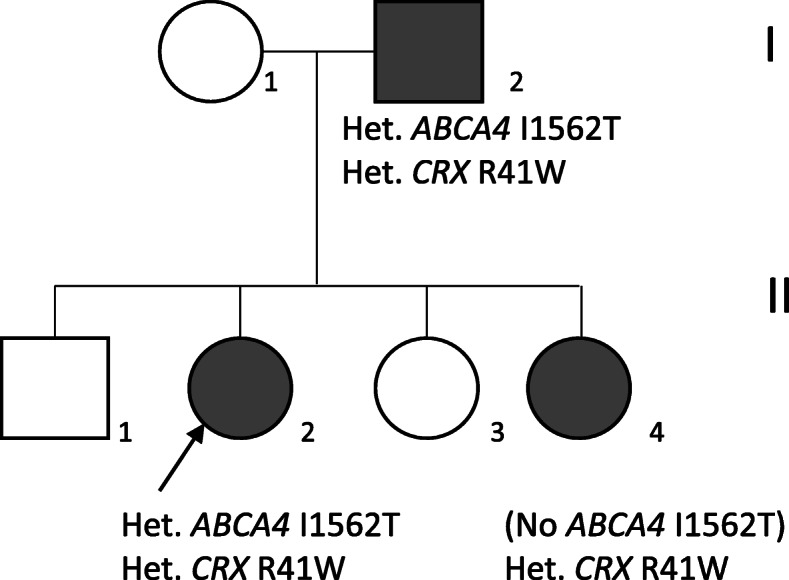
**a** Pedigree of the family. The black arrow indicates the proband II:2. The shaded boxes and circles indicate affected individuals (**b**).

**Fig. 4:**
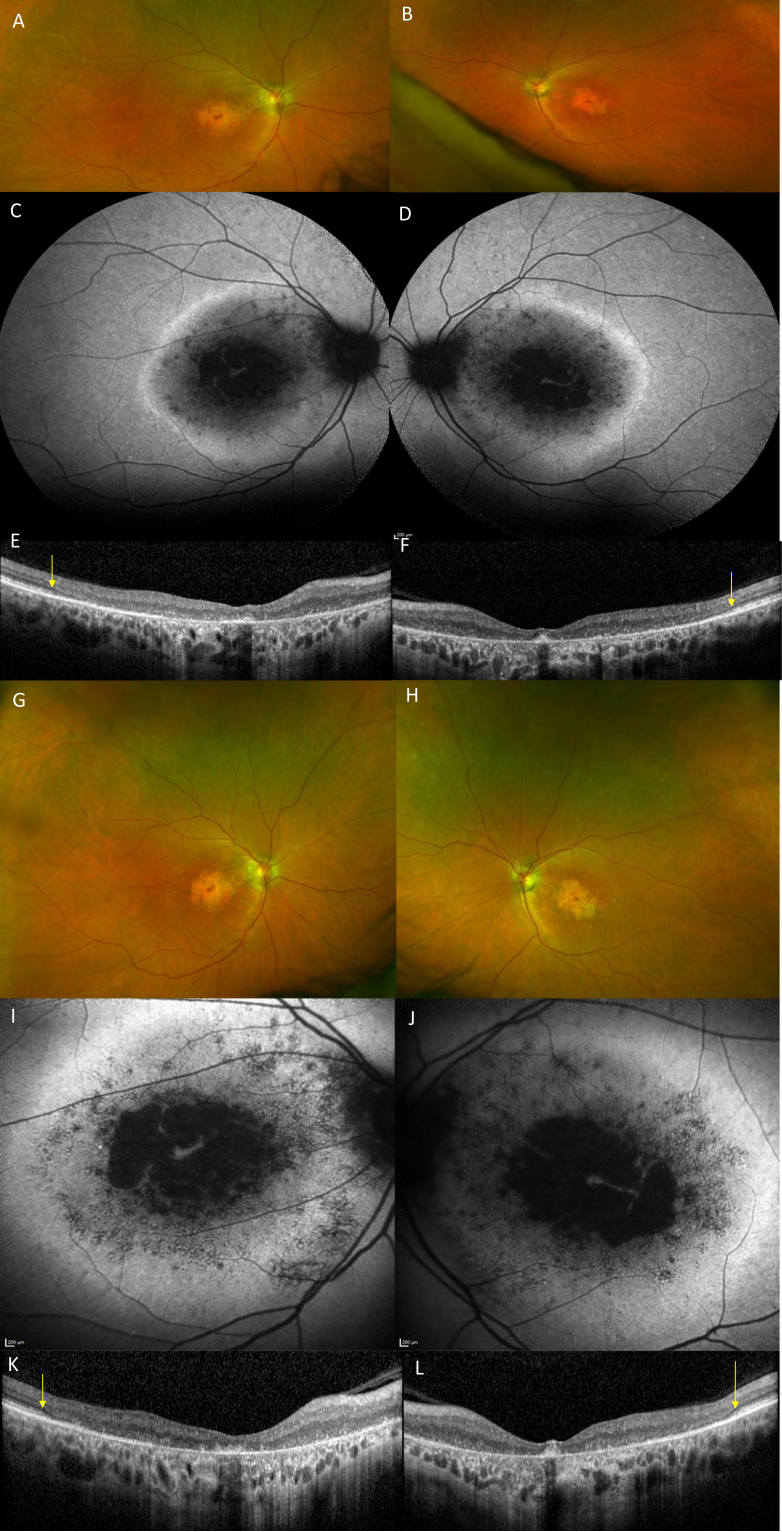
Imaging of the proband’s younger sister (II/4). Images (**a** to **f**) at review aged 56, at 38 years after diagnosis. Optos imaging showing atrophic maculae (**a** and **b**). Autofluorescence (AF) shows loss of signal centrally corresponding to atrophy with a surrounding band of increased AF within the arcades (**c** and **d**. optical coherence tomography (OCT) imaging showing the temporal extent of the loss of the ellipsoid zone demarcated by yellow arrows. **e** and **f**. Images (**g** to **l**) showing increased atrophy after a 4-year interval. Optos imaging shows increased bilateral macular atrophy (**g** and **h**). AF imaging shows extension of patchy AF surrounding the central atrophy with a small preserved foveal remnant (**i** and **j**). OCT showing further extension of the loss of the ellipsoid zone demarcated by the yellow arrows

**Fig. 5:**
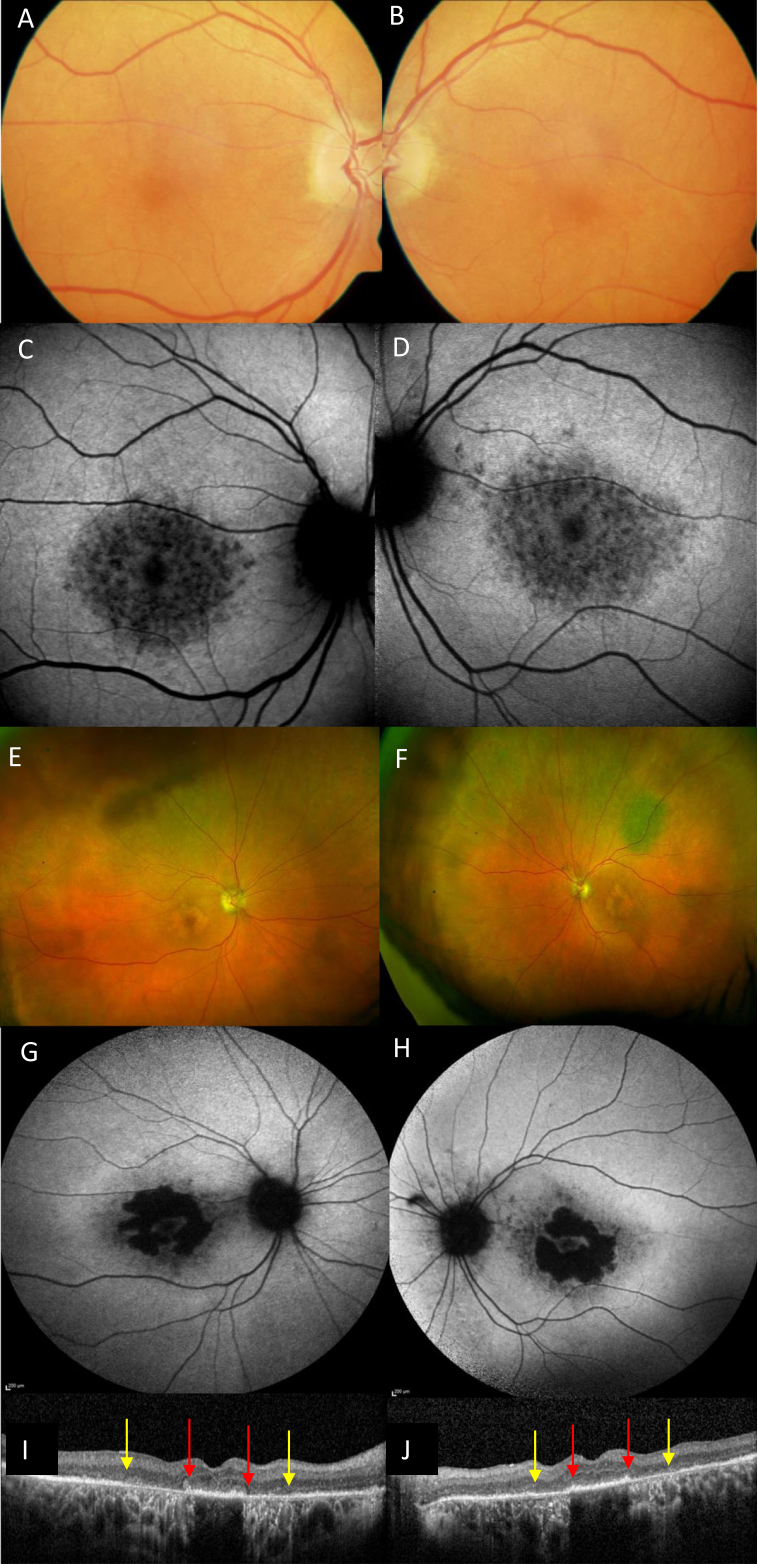
Imaging of proband’s father (I/2) Images (**a** to **d**) at age 73 (asymptomatic. Colour images of both maculae which appeared normal (**a** and **b**). Autofluorescence (AF) revealed central patchy reduction in AF signal, consistent with early atrophy (**c** and **d**). Images E to J of I:2 9 years later. Optos imaging showing distinct macular atrophy in both eyes and a choroidal naevus at left superotemporal arcade (**e** and **f**). AF imaging showing further atrophy at the central macula with central foveal sparing (**g** and **h**). Optical coherence tomography (OCT) showing preservation of the ellipsoid zone in the foveal area demarcated by red arrows, and the extent of loss of the ellipsoid zone denoted by yellow arrows (**i** and **j**)

A 43-year-old white female (II:2) presented with reduced vision in her right eye, inadvertently noticed when closing her left eye. On examination visual acuities were 6/60 OD and 6/7.5 OS. Fundoscopy revealed bilateral central atrophy, seen clearly on autofluorescence imaging (AF) (Fig. 1a and b). Eight years later AF imaging showed a preserved small central spot of AF, surrounded by a reduced AF signal corresponding to the area of atrophy, which increased in size over time, with a ring of AF. (Fig. 1c-f). Optical coherence tomography (OCT) imaging, 10 years after initial presentation, showed loss of the ellipsoid zone (EZ) (Fig. 1g and h). These features were consistent with a macular dystrophy, with features of bull’s eye maculopathy (BEM). Electrophysiology testing showed that the pattern electro-retinogram was significantly affected in both eyes; the rod electro-retinogram (ERG) showed slight increase in implicit times of both ‘a’ and ‘b’ waves with significant reduction of the ‘b’ wave amplitude. The photopic and 30 Hz flicker stimuli were normal; the EOG light rise was borderline reduced in the right eye, and normal in the left (Fig. 2). In view of the family history of Stargardt macular dystrophy (STGD1) and the BEM phenotype genetic testing for *ABCA4* variants was undertaken. Sanger sequencing was used to screen all 50 exons and approximately 50 base pairs of flanking intronic regions (standard practice at the time) in the proband. This identified a single previously reported pathogenic variant c.4685 T > C, p.(I1562T) [1], in the proband (II/2, Fig. 3), but a second variant was not identified; (at that time, 15–30% of STGD patients were reported as having only one detectable mutation in ABCA4 [2–4]). A family survey was then performed including her sister (II:4) and father (I:2), who were consented and examined. Her sister (II:4), previously diagnosed with STGD1 aged 18 years old, was re-examined at 46 years. Visual acuities were 6/60 right and left, and fundoscopy showed marked central atrophy (Fig. 4 a and b). The atrophy seen on AF and OCT imaging were similar to that seen in the proband, but were more advanced (Fig. 4c to f). Multimodal imaging at follow up 4 years later, aged 54 years, showed a further increase in central atrophy (Fig. 4g-l). Their father (I:2), aged 70 years old, was asymptomatic with visual acuities of 6/6 right, and left with a normal appearing fundus (Fig. 5a and b). However, his AF imaging showed abnormalities of central stippled reduction in signal, consistent with early macular atrophy (Fig. 5 c and d), but less advanced than that seen in his daughters (Figs. 1 and 4). Nine years after his diagnosis, aged 79, his visual acuities had deteriorated to 6/60 right, and left and he had developed central atrophic changes similar to his daughters (Fig. 5e-j). Electrophysiology results were not available for II:4, or I:II. None of the family had any co-existing systemic disorders.

Segregation analysis in the family revealed that her father (I:2) carried the c.4685 T > C variant, but the proband’s affected sister (II:4) did not, indicating that this *ABCA4* variant was not relevant to the macular dystrophy in this family (Fig. 3).

Targeted sequencing of 70 genes associated with retinal degeneration was performed in the proband [5]. A heterozygous variant, c.121C > T (p.R41W), was identified in the *cone-rod homeobox* (*CRX*) gene. Sanger sequencing confirmed the variant was present in all three affected family members (Fig. 3). This variant has previously been reported as a dominant pathogenic variant that causes cone-rod dystrophy (CRD) [6]. The family were informed of the genetic diagnosis and had follow-up genetic counselling for an autosomal dominant disease inheritance pattern associated with variable expressivity and reduced penetrance.

## Discussion and conclusions

Single *ABCA4* variants are frequently identified in patients phenotypically diagnosed with Stargardt macular dystrophy (STGD1) [2–4], *ABCA4* testing was undertaken because of the family history of STGD1 and because the siblings had a bull’s eye macular dystrophy (BEM) appearance, which has been reported with *ABCA4* variants [7]. However once the family survey was performed, with no *ABCA4* variant identified in II/4, and a macular dystrophy diagnosed in her asymptomatic father (I:2), an *ABCA4* retinopathy was excluded. The gene panel testing identified a pathogenic sequence variant in *CRX.* The *ABCA4* variant was designated to be an incidental finding (carrier status) (Fig. 3).

The *CRX* (*cone rod homeobox*) gene was first identified as an important regulator of photoreceptor cell development by Chen et al. in 1997 [8]. Then Swain et al. and Freund et al. reported that *CRX* caused autosomal dominant cone rod dystrophy (CRD) [6, 9]. Swain et al. first described the c.121 T > C variant with a similar phenotype to that seen in the family we report here, except that their case in addition to rod degeneration also had cone dysfunction, which was not seen in the proband in our family [6]. A recent study by Nishiguchi et al. highlighted a range of phenotypes, observing that *CRX* mutations occurring downstream in the homeobox domain (p.L299F, p.G243X, p.S204fs and p.S213fs) had diffuse or posteriorly extending bone-spicule pigmentation and vascular attenuation consistent with retinitis pigmentosa whilst all their other patients with mutations in the homeobox domain had a phenotype that was consistent with a macular dysfunction or CRD typified by a central region of decreased AF surrounded by a ring of increased AF and loss of the EZ in the maculae of all the symptomatic CRX patients [10]. In addition to variable expressivity, the Nishiguchi et al. study highlighted reduced penetrance; two of the families with the *CRX* variant p.R41W had affected parents with a milder phenotype than their affected children. This is similar to the father I:2 in our family, who was asymptomatic when examined at the family survey. The visual acuity level in their cohort was similar to the VA seen in our 3 patients [10]. Three studies report an electronegative ERG with some *CRX* mutations [6, 10, 11]. Cone dystrophy alone, as seen in the proband in this family has been previously described [12] and Hull et al. describe a rod cone dystrophy phenotype associated with this same *CRX* c.121C > T variant [11].

Intra and inter-familial variability of expression and reduced penetrance seem to be a hallmark of *CRX* [10–15]. Romdhane et al. report intra-familial variability and reduced penetrance, as seen in this family and a BEM phenotype [15]. BEM associated with *CRX* sequence variants has been reported by a number of authors, characterised by a central decreased AF signal surrounded by a ring of increased AF and loss of the EZ on OCT imaging and foveal preservation [11, 16]. It is clear however that the presence of BEM is not pathognomic for *CRX*, as BEM has been described in association with sequence variants in *CRX*, *ABCA4* and *GUCY2D* [16]. The spared fovea has been seen in patients with *CRX* mutations before, with Boulanger-Scemama et al. reporting that half of their cohort of *CRX* patients had a spared fovea [17].

*CRX* variants have been described in association with Leber congenital amaurosis and rod cone dystrophy, in addition to the typical cone rod/macular dystrophy phenotype [11].

More recently subsequent to the testing identifying the *CRX* variant in this case, Wollock et al. reported on the use of genome wide enrichment analysis of qualifying variants for dominant inheritance pattern diseases using the collapsed analysis approach. This type of approach reached a study wide significance for identification of *CRX* mutations in patients that were clinically diagnosed with STGD1, CRD, BEM, PD and MD, but were not found to have mutations in the *ABCA4* gene thus highlighting the importance of using a multiple gene panel when assessing these patients [18].

In summary this family study illustrates the complexity of genetic testing in the context of phenocopies, reduced penetrance and variable expressivity. This is illustrated by the discovery of a clinical phenotype in the asymptomatic father using multimodal imaging, revisiting the original diagnosis in the sister, and showing non segregation of the *ABCA4* variant, as well as identifying the pathogenic *CRX* variant in all three affected family members using NGS. It highlights the importance of comprehensive genetic testing and a careful family survey. If a family survey had been performed at the initial visit, the asymptomatic father would have been identified as affected, and an autosomal dominant pattern of inheritance with segregation would have excluded *ABCA4* as a causative gene. However, it is not always possible to perform a family survey, which can make interpretation of genetic testing results difficult in cases where more than one variant is identified as potentially causative and where there are known phenocopies.

With the advent of whole genome analysis many potential pathogenic variants will be identified in different genes; accurate phenotyping and family studies will be key in identifying the variant(s) in causative genes, and will enable diagnosis in phenocopies and complex phenotype-genotype correlations. This case highlights the variable expression and reduced penetrance seen in *CRX* associated retinal dystrophies. It also emphasises the value of access to NGS genetic analysis and the importance of family studies with segregation to complement and direct genetic testing.

## Data Availability

All data generated or analysed during this study are included in this published article.

## Abbreviations

ABCA4: ATP binding cassette subfamily A member 4
AD: Autosomal dominant
AF: Autofluorescence
AR: Autosomal recessive
BEM: Bull’s eye maculopathy
CRD: Cone-Rod dystrophy
CRX: Cone-rod homeobox
OCT: Optical coherence tomography
STGD1: Stargardt disease
